# Slurring of Speech and Lip Paresthesia: Symptoms of Levodopa End of Dose Wearing Off in Parkinson's Disease

**DOI:** 10.7759/cureus.2986

**Published:** 2018-07-16

**Authors:** Usama Nasir, Muhammad Ali Tariq, Asad Ali, Syed Daniyal Asad, Ahmer Asif, FNU Farukhuddin, Rovaid Khan, Haris H Chaudhry, Sarah Choudry

**Affiliations:** 1 Medicine, CMH Lahore Medical College and Institute of Dentistry, Lahore, PAK; 2 Angiocore Lab, Medstar Washington Hospital Center, Washington, USA; 3 Internal Medicine, Lahore Medical and Dental College, Lahore, PAK; 4 Neurology, University of Missouri Hospital, Columbia, USA; 5 Cardiology, CHI Baylor St. Lukes, Houston, USA; 6 Medicine, Ayub Medical College, Peshawar, PAK; 7 Surgery, University of California, Irvine, Orange, USA

**Keywords:** parkinsons, levodopa, on-off phenomenon, tia, slurring of speech

## Abstract

The prolonged use of levodopa for treating Parkinson’s disease is associated with motor and nonmotor complications. These include wearing-off, delayed-on, partial-on, no-on, and on-off phenomena. In the wearing-off effect, symptoms return before a patient's next scheduled dose of levodopa. Patients may present with motor, sensory, or autonomic fluctuations. In this report, we present a female patient experiencing numbness of lips and slurred speech as a symptom of wearing-off effect. The major differential for sudden numbness of lips and slurred speech includes transient ischemic attacks. Therefore, it is imperative to identify the cause of these episodes so that appropriate treatment can be initiated. Our patient underwent extensive cardiac and neurological investigations, the findings of which were unremarkable. Her symptoms were likely due to levodopa wearing-off. Her condition improved on changing her levodopa to a sustained release form with more frequent dosing along with the addition of ropinirole to her treatment regimen.

## Introduction

Parkinson’s disease (PD) is the second most prevalent neurodegenerative disorder [1], and it affects 1% of the population above 60 years of age [2]. The pathophysiology is explained by a loss of dopaminergic neurons in the Substantia Nigra, manifesting as a paucity of voluntary movements. Therefore, therapy is aimed at restoring this dopamine deficiency. Treatment options in the early stages of the disease include dopamine receptor agonists, anticholinergics, MAO-B inhibitors, amantadine, etc. However, with the progression of PD, patients require levodopa for effective symptom management [3].

Initiating levodopa treatment is usually followed by an initial “honeymoon period” where levodopa almost completely reverses the signs and symptoms of PD [4]. However, with disease progression, the majority of patients start to experience a gradual shortening in the duration of levodopa’s beneficial effect, and the “wearing-off” phenomenon. Wearing off can be described as the recurrence of PD symptoms before the normal dosing cycle of levodopa has ended [3]. During the wearing-off period, patients may present with sensory, autonomic, motor, and psychiatric symptoms. Other complications that might be seen during ‘off’ periods are paresthesia, pain, tachycardia, sweating, double vision, numbness, belching, constipation, and dyspnea [5-6].

Here, we document an elderly female from Pakistan who has been on levodopa therapy for the past eight years and now presented with recurrent numbness of lips and difficulty in speaking.

## Case presentation

An 84-year-old right-handed female was diagnosed with PD at the age of 75. Her initial symptoms included bradykinesia, rigidity, and resting tremor of the right hand. The patient was initially started on levodopa/carbidopa 250/25 mg twice daily, which resulted in a significant improvement of her symptoms. Levodopa dosage was gradually increased over the years to levodopa/carbidopa 250/25 mg, four times a day. After a total of eight years on levodopa therapy, the patient started experiencing numbness of lips, along with difficulty in speaking approximately 45 minutes before the next scheduled levodopa dose. The patient described these episodes as sudden numbness, predominantly of the upper lip and a ‘feeling of the lips becoming heavy.’ This usually progressed within minutes to difficulty in speaking and freely opening the jaw. Neurological examination during these episodes showed an alert, awake, and oriented patient with difficulty in pronouncing both monosyllabic and polysyllabic words. Sensations to fine touch were decreased on the upper lip but were intact bilaterally on the rest of the face as well as the body. The patient’s anxiety and frustration over these symptoms resulted in a visit to the emergency room. The possibility of transient ischemic attack (TIA) was ruled out with a detailed neurological consult including brain and carotid imaging. A cardiovascular workup was also done which was unremarkable. Physical examinations during all these episodes showed an anxious patient, with difficulty in speaking and generalized decreased motor strength throughout the body.

After a careful review of history and discussion of the case with her primary doctor, a diagnosis of levodopa’s “wearing off” phenomenon was made. The patient consulted her neurologist with respect to the symptoms and was put on a controlled release form of levodopa with the addition of ropinirole. The new regimen successfully ameliorated the wearing-off phenomenon. Interestingly it was noted that delaying the scheduled dose of levodopa would cause the return of the symptoms.

## Discussion

Patients of PD on prolonged levodopa therapy frequently suffer from “end of dose or wearing-off symptoms” (EODWO). According to Nutt et al. [7], nearly 50% of all patients will experience one or more of these symptoms after an average of five to six years of levodopa use. These symptoms are fairly diverse in presentation and include a variety of motor, sensory, autonomic, and psychological abnormalities.

Numbness of lips and speech difficulty in an elderly patient may lead to a workup for TIA. Patients may undergo investigations, such as computed tomography (CT) brain, carotid Doppler, echocardiography, and electrocardiograms (EKGs) to identify the cause of the symptoms. However, careful history taking may allow physicians to relate the levodopa dosing schedule to the presenting complaints. A thorough literature review concluded that these symptoms have not been documented previously and so this report is the first of its kind. The speech changes in PD include but are not limited to monopitch, reduced overall loudness, short rushes of speech, short phrases, variable rate, overall increased rate, inappropriate silences, repeated phonemes, reduced stress, and imprecise consonants [8]. Perhaps, the pathophysiology of the slurred speech experienced by the patient stems from the same roots as levodopa’s effects start to wear off towards the end of the dose.

Strategies for managing wearing-off phenomena primarily focus on adjusting the drug dose for optimal effect. There are several options that may be successful in achieving this outcome, such as shortening the levodopa dosage interval, using catechol-O-methyl transferase (COMT) inhibitors or dopamine receptor agonists [6].

The patient was started on ropinirole, a dopamine receptor agonist with the aim of gradually decreasing the dose of levodopa. In addition to this, the patient’s regular levodopa was changed to a controlled release form with the formulation of 200/50. The dosage regimen being followed was ropinirole 0.5 mg three times daily and levodopa controlled release 200/50 every six hours. With the controlled release therapy, the plasma concentration of levodopa may be maintained at sufficient levels so as to prevent the manifestation of wearing-off symptoms for over eight hours [9]. Lees reported that patients on controlled release therapy may require considerably larger doses of levodopa [9]. Hauser (2004) and Hauser et al. (1998) support adding a COMT inhibitor such as entacapone or a monoamine oxidase-B inhibitor such as selegiline to alleviate the EODWO phenomenon [10-11]. Both entacapone and selegiline prevent the enzymatic degradation of levodopa, thereby prolonging levodopa's half-life and delaying the onset of the EODWO.

The management of wearing off is dependent on the early identification of symptoms coupled with the early initiation of effective treatment. Major issues here include the need to educate the patients and their caregivers in addition to facilitating good communication with primary and secondary healthcare professionals.

## Conclusions

In conclusion, patients on prolonged levodopa therapy can present with certain sensory, motor, or autonomic symptoms before the next scheduled dose of levodopa. Slurred speech and lip paresthesia as symptoms of levodopa EODWO in PD are a rare scenario. We are unable to explain the precise pathophysiology for these symptoms but it will be interesting to follow future cases that are similar in presentation to compare and contrast them with this case.
